# Pediatric Health, Climate Perceptions, and School Absenteeism Across Three Regions of Bangladesh: A Cross-Sectional Study

**DOI:** 10.3390/ijerph22111639

**Published:** 2025-10-28

**Authors:** Yoon Sik Jung, Sakila Afroz, Sadia Samad Mow, Xingyan Wang, Caroline Sarpy, Md Fuadul Islam, Md Nazmul Husain, Md Shahadat Hossain, Al Romana Sania, Md Golam Mostofa, Quazi Quamruzzaman, Maitreyi Mazumdar

**Affiliations:** 1 Department of Environmental Health, Harvard T.H. Chan School of Public Health, Harvard University, Boston, MA 02115, USA; yjung2@hsph.harvard.edu; 2 Dhaka Community Hospital Trust, Dhaka 1217, Bangladesh; joya9878@yahoo.com (S.A.); mou.sadia08@gmail.com (S.S.M.); fuadislam1996@gmail.com (M.F.I.); nazmuldcht@gmail.com (M.N.H.); shahadat.dcht@gmail.com (M.S.H.); romanasania2020@gmail.com (A.R.S.); mostofa07@gmail.com (M.G.M.); qzaman07@gmail.com (Q.Q.); 3 Department of Epidemiology, Harvard T.H. Chan School of Public Health, Harvard University, Boston, MA 02115, USA; xingyanwang@hsph.harvard.edu; 4 Department of Neurology, Boston Children’s Hospital, Boston, MA 02115, USA; caroline.sarpy@childrens.harvard.edu

**Keywords:** climate change, public health, Bangladesh, environmental health, environmental exposure, blood lead, epilepsy, school absenteeism

## Abstract

Children remain underrepresented in environmental health studies, and evidence on how climate-related exposures affect pediatric health and school absenteeism is limited. This pilot cross-sectional study reports pediatric symptoms, school attendance, and perceptions of climate change among 300 Bangladeshi children ages 6–12 years old in three sites: Barhatta, Galachipa, and Sarankhola. Health status, climate-related perception, and educational disruption were assessed with validated questionnaires. Clinical staff measured peak expiratory flow rate, hemoglobin, and blood lead concentrations. Rash (48%), asthma (21%), and positive screening for epilepsy (17%) were most prevalent in Sarankhola. Mean hemoglobin was lower in Sarankhola (11.0 g/dL) than in the other sites. Awareness of climate change was 100% in Galachipa and Sarankhola, while 32% in Barhatta, with television and health workers being the common sources of information. Almost one in every three children missed at least three days of school in the last month with illness, climate-related emergencies, and unexpected school closures being frequent causes. These findings indicate that Bangladeshi children, especially those living in coastal areas, face the health and educational risks related to climate change, and that longitudinal and environmental monitoring studies are needed.

## 1. Introduction

As global temperature continues to rise at a record pace, climate change heavily burdens the ecosystems and the health of individuals [1,2]. Children’s health is at high risk due to climate change [3]. Biological factors such as narrower airways, developing nervous and immune systems, and higher air, water, and food intake per body mass make children highly vulnerable [4,5]. A recent model published in Nature concludes that children born in the 2020s are far more likely to face extreme lifetime heat exposure than those born in 1960s; at least 52% of people born in 2020 will experience exposure to heatwaves, and the chance of heatwave exposure is substantially larger among population groups characterized by social and economic vulnerabilities [6].

Systematic reviews have demonstrated that climate events are closely linked with the aggravation of mental health disorders, respiratory illness, diarrheal illness, vector-borne disease, malnutrition and stunted growth rates, disruption of caregiving, and income loss [7]. Rising temperature has been associated with elevated childhood dehydration, asthma exacerbations, and vector-borne illness. Climate change may also redistribute environmental contaminants, such as heavy metals, that affect neurodevelopment [8]. There are fewer studies that investigate the effects of climate change on learning outcomes and educational attainment. Reduced academic performance and poor lung function are associated with air pollution [9,10]. Floods, heatwaves, and cyclones are extreme weather events that undermine access to healthcare and education, displace households, and introduce instability disproportionately experienced by children [11]. However, children are still underrepresented in environmental health research. Much of the research considers adults or does not stratify outcomes by age, diminishing the relevance to pediatric populations [12].

In addition to the direct health effects in children, there is increasing awareness that climate change may affect school attendance and learning. Climate change may exacerbate illnesses in children as well as keep a child home from school for care of other children or family members [13]. Climate-oriented mental health challenges such as anxiety and bereavement affect children as well as adults, and mental health problems in children have long been linked to school attendance problems [14,15]. In the Global South in particular, climate change has produced mass migration, which often leads to intermittent school attendance and premature withdrawal from school [16].

Bangladesh is severely affected by climate change with events like heat waves, saltwater intrusion, sea-level rise, and flooding [17,18]. With most impact on its low-lying coastal areas and highly populated cities, around 13 million Bangladeshis are estimated to be displaced by 2050 [19]. In most riverine and coastal communities, residents experience simultaneous exposure to saline water consumption, polluted air, and hindrances in accessing school or health services following climate-related activities [20,21]. Rural communities are at greatest risk, yet there are few studies investigating how the environment influences an area of health across several domains in these communities. Prior studies have either been based on a single outcome or non-standardized measures, making comparison or intervention design difficult. Because flooding, salinization, and industrial runoff can redistribute heavy metals such as lead, we included blood lead measurement as an indicator of environment exposure relevant to climate-related contamination in children [22].

**Figure 1:**
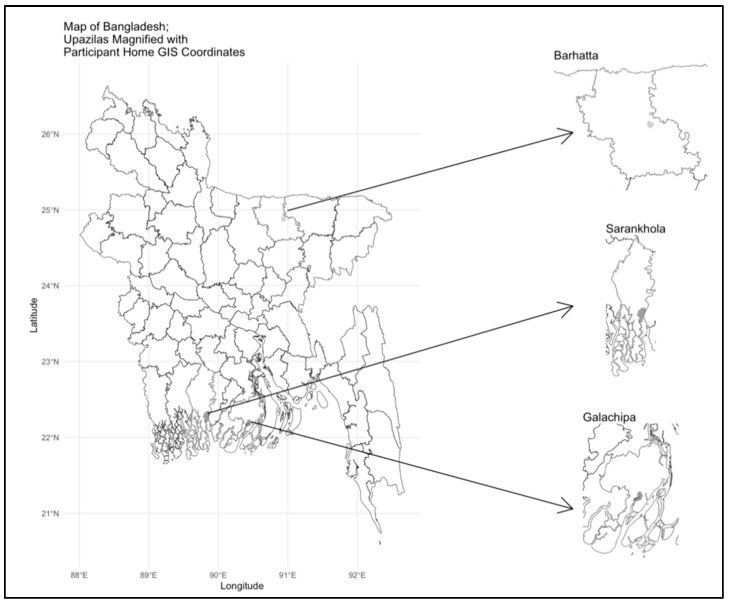
Location of study sites.

The objective of this cross-sectional study, therefore, was to describe pediatric health indicators, climate change perceptions, and school absenteeism among children in the age group of 6–12 years (i.e., school-age) in three rural locations in Bangladesh (Figure 1), using standardized questionnaires and clinical measures. Children aged 6–12 years were selected because this group represents the primary school-age population, a period marked by rapid physical and cognitive development and consistent school attendance [23]. These characteristics of this age range make it ideal for examining the link between pediatric health, school absenteeism, and climate-related exposures.

We selected three sites, Barhatta of Netrokona District, Galachipa of Patuakhali District, and Sarankhola of Bagerhat District (Figure 1). We selected these sites because they had strong relationships with Dhaka Community Hospital Trust. Galachipa and Sarankhola are coastal upazilas, which experience more tidal flooding, saline intrusion, and cyclones, while Barhatta is an inland upazila, which is in a floodplain with different environmental and infrastructural characteristics. These sites were chosen to enable exposure variation to geography and regional disparities in health and education within Bangladesh. We present descriptive statistics to demonstrate and guide the development of future studies in these settings.

## 2. Materials and Methods

### 2.1. Study Population

Three hundred children aged 6–12 years were surveyed between August and October 2024 in three clinics run by Dhaka Community Hospital Trust in Bangladesh. A cross-sectional design was selected for this pilot study to capture baseline health and environmental exposure data from multiple regions within a short time span.

We recruited 100 children at each site to provide balanced representation across regions. We approached families in the clinic waiting room with a recruitment script that described study activities. Parents provided written consent for their child’s participation. Verbal assent was obtained from all participating children. Inclusion criteria were children aged 6–12 years who attended one of the three participating Dhaka Community Hospital Trust clinics during the study period and whose parents or guardians provided written informed consent. Exclusion criteria were children outside the specified range and those whose parents declined participation.

All protocols were reviewed and approved by the institutional review boards of Boston Children’s Hospital (BCH) (Protocol number: IRB-P00047958) and Dhaka Community Hospital (DCH). Participants received school supplies as a gift for participation.

### 2.2. Questionnaires

At the clinic, trained study staff including medical assistants and fieldworkers employed by Dhaka Community Hospital Trust administered the following validated and standardized questionnaires: Climate Questionnaire [24], Epilepsy Screening Questionnaire [25], General Health Questionnaire [26,27,28], and School and Absenteeism Questionnaire [29,30]. These structured questionnaires have been validated to collect data on school attendance, medical and family histories, climate change awareness, and exposure to climate change events. All questionnaires were originally developed in English and were professionally translated into Bangla prior to administration to ensure participant comprehension. Caregivers mainly provided responses to the questionnaires with assistance as needed.

Participants were considered to have screened positive for epilepsy if their total screening score was greater than 1. The screening tool included 10 items, with any “Yes” response to items Q1–9 triggering a specialist review. If only Q10 was endorsed, further discussion determined need for review.

The School and Absenteeism Questionnaire was adapted from previously validated sources, including WHO’s Global School-based Student Health Survey and a prior climate perception study conducted in Bangladesh. Participants were asked how many school days they missed over the past 30 days and the reasons for those absences, with multiple options available, such as illness and transportation.

### 2.3. Physical Examination

Trained staff measured each participant’s height (cm) with a portable stadiometer, weight (kg) with a scale, and mid-upper arm circumference (MUAC, cm) using a tape measure, following a standardized protocol. Peak expiratory flow rate (PEFR) was measured in liters per minute (L/min) using BSMI peak flow meters (Bismillah S.M. Industries, Dhaka, Bangladesh).

### 2.4. Blood Lead and Hemoglobin Concentration

We measured blood lead concentrations using point-of-care testing from capillary blood samples using LeadCare II^®^ (Meridian Bioscience, Inc., Billerica, MA, USA), an FDA-approved and validated instrument for blood lead screening [31]. We determined hemoglobin concentration using the Sahli hemoglobinometer method. The limit of detection (LOD) for lead was 3.3 µg/dL. “Low” lead results in the display window were treated as 1.65 µg/dL, half of the limit of detection. Both devices were calibrated daily according to manufacturer guidelines.

### 2.5. Water Salinity and GIS Coordinates

Water salinity was selected as environmental indicators given their known sensitivity to climate-driven changes in water quality and soil contamination in coastal Bangladesh [32]. Within a few days of the clinic visit, study staff visited each participant’s home and collected two water samples: the first from the primary drinking water source and the second from cooking or bathing source. Salinity was analyzed at DCH and Bangladesh University of Engineering and Technology (BUET) laboratories using a HACH sensION5 Electric Conductivity meter (HACH, Loveland, CO, USA). The detection limit was 0.1 parts per thousand (ppt). Data was recorded numerically in ppt or below the method detection limit (>MDL). Geographic coordinates for the participants’ homes were recorded using a handheld GPS device (GPSMAP^®^ 62, Garmin Ltd., Olathe, KS, USA).

### 2.6. Statistical Analysis

All questionnaire and clinical data initially collected using paper forms were entered into a secure electronic database and verified by cross-checking for accuracy. Environmental measurements and laboratory results were linked to participant data using unique identification codes. Only de-identified, coded data were used in analysis.

Data management was conducted using Microsoft Excel, and statistical analyses were performed in R (Version 2025.05.0 + 496; R Foundation for Statistical Computing, Vienna, Austria). Descriptive statistics were used to summarize the data including means and standard deviations for continuous variables, medians and IQR for skewed variables, and frequencies with percentages for categorical variables.

## 3. Results

### 3.1. General Characteristics of the Study Population

Out of 320 households approached, 24 declined participation. Reasons for refusal were not having sufficient time, discomfort with blood sampling, or refusal to participate in the home visit. The final enrolled sample comprised 300 children with 100 per site.

Demographic and household characteristics across the three study sites are presented in Table 1. Most of the household heads were male, and the most frequent head of household occupations were farmer and day laborer. Patterns of reported child health conditions differed across sites. Sarankhola recorded a much higher prevalence of asthma (*n* = 21), epilepsy (*n* = 17), and rash (*n* = 48). Age-specific distribution showed that ages 9–10 had the highest epilepsy prevalence in Sarankhola (Supplementary Table S1).

Blood lead measurements (Table 2) revealed detectable levels of lead in most children, although in amounts below the threshold of potential harm to health. Median blood lead concentrations were 4.60 µg/dL in Barhatta, and below the level of detection in both Galachipa and Sarankhola.

### 3.2. Climate and Health-Related Perceptions

As shown in Table 3, the major source of drinking water was shallow water in Barhatta and deep tubewell in Galachipa. Sarankhola observed a slight majority of rainwater as drinking water source and was overall more diverse than Barhatta and Galachipa in their drinking water sources. Healthcare access was primarily from NGO Healthcare Center in Barhatta and government hospitals in Galachipa and Sarankhola. In Sarankhola, 78% had experienced homelessness due to natural calamity in the past decade, compared to 4% in Barhatta and 41% in Galachipa. Only 10% of children in Barhatta indicated a health education program exist in their school, compared to 98% in Galachipa and 93% in Sarankhola. All 200 children in Galachipa and Sarankhola reported hearing about climate change, while the number was only one-third in Barhatta. All three sites reported the most common perceived type of climate change as excessive temperature.

### 3.3. Household Water Salinity

Table 4 summarizes salinity measurements based on home visits and perceptions based on questionnaires. Median salinity measures for drinking and cooking water were higher in Galachipa, with most of the measurements in Sarankhola below or near detection threshold of 0.05 ppt. Although all three sites fell below the commonly used 0.5 ppt criterion for freshwater, Galachipa’s higher median with lower range suggests consistently higher baseline salinity. None of Barhatta’s households reported a perceived increase in local water salinity over the past 10 years, while 89% of both Galachipa and Sarankhola reported a perceived increase in local water salinity.

Most households in Barhatta and Galachipa used the same water source for drinking and bathing or cooking, (96% and 98%, respectively) as compared to fewer than half in Sarankhola. Although measured values fell within the freshwater range, the general impression of worsening salinity, particularly in Galachipa, could be an indication of long-term exposure tendencies or issues not defined by isolated measurements.

### 3.4. School Absenteeism

Table 5 shows that almost one-third of children in each site missed at least three days of school in the prior 30 days. Barhatta reported high rates of unexpected school closures (*n* = 39), Galachipa reported high rates of personal illness (*n* = 36) and family illness (*n* = 16), and Sarankhola reported high rates of personal illness (*n* = 48) and weather or climate emergency (*n* = 25). Regarding perceived climate-related impacts on education, 58% of children in Barhatta reported being unaffected, compared to 20% in Galachipa and 23% in Sarankhola. The most common disruption in Galachipa was difficulty traveling to school (71%); in Sarankhola, 61% reported the same issue.

## 4. Discussion

This study adds to a growing number of studies assessing health conditions associated with climate change among people in Bangladesh as well as perceptions of climate change among families. The participants in this study were children and their families in Galachipa, Sarankhola and Barhatta who received care in health centers served by Dhaka Community Hospital Trust, a private, non-profit organization established in 1988 to provide healthcare for low-income families in both urban and rural areas of Bangladesh. These areas were chosen because of strong ties to the health center and differences in exposures. Galachipa and Sarankhola are both coastal, low-lying upazilas on the Bay of Bengal with high exposure to tidal flooding, saline intrusion, and cyclones [33,34]. Barhatta is an inland upazila in Netrakona District, located in a floodplain and wetland region with greater population density, enhanced road and railroad connectivity, and relatively more urban facility access [35].

This study utilized validated and standardized tools and surveys to ensure reliable and comparable data collection across sites. The findings from these instruments provide insight into both the physical and psychosocial dimension of child health in Bangladesh. For example, reported respiratory symptoms and absenteeism patterns corresponded with local environmental conditions, while responses to Climate Questionnaire highlighted growing awareness of environment change among families.

We found that children aged 6–12 in the coastal areas of Galachipa and Sarankhola reported greater awareness of climate change, with 100% of participants reporting hearing about climate change. In Barhatta and Sarankhola, the most frequently cited source of information was television. In Galachipa, health workers were cited as the primary source. These trends also suggest that awareness of climate change comes not only from experience but also from more assertive communication by schools, health services, and communities.

In Sarankhola, for example, 78% reported homelessness from extreme weather, and 61% reported journey to school was impacted by climate-related disturbances. Across all sites, 32% of children missed ≥3 days of school in the past month, and illness and extreme weather were the most common reasons. Children in Sarankhola also had high rates of asthma (21%) and rash (48%), both conditions potentially caused or exacerbated by warmer temperatures and pollution.

Large differences in health indicators were also observed across sites. Sarankhola had a notably higher rate of children screening positive for epilepsy, with 17 out of 20 total positive screens (85%) coming from this site. While some variation may reflect measurement or reporting differences, the concentration of possible epilepsy in one site warrants further clinical follow-up. Mean hemoglobin in Sarankhola (11.0 g/dL) was lowest, contrasting with Galachipa (13.1 g/dL) and Barhatta (12.8 g/dL). In comparison, other population-based data show a mean hemoglobin of 13.5 g/dL in healthy children aged between 6 and 12 years, with 11.5 g/dL as the two standard deviations below the mean [36]. The lower mean in Sarankhola may suggest a higher burden of nutritional deficiencies (such as iron), inflammation, or environmental exposures affecting child health [37].

This study contributes to the body of literature by focusing on children, a population that bears about 88% of the world’s climate-related health burden [38]. Children are more physiologically susceptible to environmental exposures and are more likely to be at risk of social disruptions, including missed school or displacement [39].

Most prior environmental health research in Bangladesh has focused on adult populations [40,41]. We also add data on climate-related school disruption, a rarely studied outcome in this context. Our findings underscore the need for child-specific assessments in climate research, especially in regions on the frontlines of climate-related displacement and environmental change.

The results of this study align with existing research. Climate change has widely impacted human health, interrupted essential public health services such as sanitation, and heightened infectious as well as chronic diseases across the world [42,43]. Climate change affects human health in Bangladesh in several ways, with the most vulnerable communities suffering high rates of malaria, dengue, diarrhea, and pneumonia [44].

A study conducted in similar rural areas established that frequent flooding, riverbank erosion, and crop damage were the primary drivers of displacement in Saghata and Fulchari upazilas [45]. Using propensity score techniques, one recent study in flood-affected areas in Bangladesh estimated infant death in households residing in flood-affected areas was 5.3 deaths per 1000 births higher than in those in lower flood-exposed areas (95% CI: 2.2–8.4) [46].

Children are at higher risk of climate health impacts because their bodies are in the process of development and are less resilient to environmental stress [3,47]. For example, projected 1.5 °C to 2 °C warming can raise hospitalizations by diarrhea up to 9.4% among children in Bangladesh, more than any other age group, due to children being more climate-sensitive [47]. There remains limited literature of pediatric epilepsy in Bangladesh, yet neurological disease is one of three top reported health conditions due to climate change (preceded by respiratory and cardiovascular diseases) [43].

Furthermore, including blood lead screening allowed us to assess an established environmental health risk that may contribute to neurodevelopmental outcomes and school performance. The presence of measurable blood lead concentrations underscores the continuing need for integrated environmental and educational health interventions in rural Bangladesh.

There are several limitations to the study. The cross-sectional design and small sample size do not permit assessment of less common outcomes, changes over time, or causation. Additionally, we recruited participants when they visited clinics, potentially selecting healthier, and more healthcare-seeking families. We also recognize that this is a convenience sample only and may not be representative of the whole population. Finally, the environmental testing revealed large number of values near or below the detection limit, which can bias exposure estimates because the tester’s equipment was less sensitive.

In our sample, around 7% of children screened positive for epilepsy, a figure considerably higher than the estimated national prevalence rate of 8.2 per 1000 among another group of children in a population-based survey [48]. While that national estimate is derived from clinically validated diagnoses across all regions, our higher screening rate could uncover undiagnosed or unrecognized illnesses in a high-risk rural pediatric population. This result suggests a potential underestimation of epilepsy burden in normal surveillance and a need for more pediatric neurologic screening in climate-affected areas. However, the considerably higher positive epilepsy prevalence could be a result of the questionnaire screening method compared to using more robust tests such as electroencephalography (EEG) or brain imaging.

This study has several strengths. It shows that it is possible to collect clinical, environmental, and survey data in low-resource, high-risk settings using standardized tools and home-based follow-up. The study is among the first to use a combination of environmental sampling, child health indicators, and climate perception data in a population level effort.

These findings can be a useful model for future, larger-scale longitudinal studies. For instance, epilepsy management in Bangladesh is especially at risk, and children who suffer from prolonged seizures often lack an emergency response plan because of economic constraints [49]. This highlights how economic vulnerability might affect neurological risk especially among the underprivileged. Furthermore, the findings suggest that health authorities should strengthen pediatric health surveillance systems such as by integrating environmental monitoring into school health programs. Health resources and interventions should be prioritized in coastal and flood-prone regions to mitigate risks identified in this pilot study.

Subsequent research must be directed towards clinical validation of neurologic outcomes, spatial analysis for merging climate datasets with health risk, and qualitative studies with families to determine adaptation practices for school absenteeism. Future larger studies should apply multivariable regression and longitudinal designs to estimate associations between environmental exposures and pediatric outcomes and consider causal inferences where appropriate. As the effects of climate change on health risks increase across the globe, underscoring children in research and response activities is crucial in high-risk countries like Bangladesh.

## 5. Conclusions

This pilot study provides insight into child health and climate risk at three sites in Bangladesh. Children in the coastal areas of Galachipa and Sarankhola experienced more frequent incursions by weather-related events, higher rates of school absenteeism, and more respiratory symptoms than children at the inland site of Barhatta. Most children have heard of climate change through television and conversations with community members or health workers.

At Sarankhola, the percentage of children screening positive for epilepsy was greater, and the average hemoglobin was lower than at the other two sites. These findings may represent increased susceptibility to environmental threats because of underlying nutritional status. The results reinforce the importance of active monitoring of climate change and the benefits of further large-scale studies to confirm these patterns and guide future healthcare strategy.

## Figures and Tables

**Table 1:** Demographic and Household Characteristics of Study Population by Site (*n* = 300).

	Children in Three Study Sites (*n* = 300)		
Characteristics	Barhatta (*n* = 100)	Galachipa (*n* = 100)	Sarankhola (*n* = 100)
Age (years)	8.7 ± 1.9	8.8 ± 2.1	9.1 ± 1.8
Sex			
Male	44	43	48
Female	56	57	52
BMI (kg/m^2^)	14.0 ± 2.8	15.1 ± 2.2	14.4 ± 1.7
Mid-Upper Arm Circumference (cm)	18.2 ± 2.6	18.5 ± 2.5	18.1 ± 2.2
Education Level			
No Institutional Education	4	0	2
Primary Level (Class 1–5)	90	88	89
Secondary Level (Class 6–10)	4	7	9
Other/Not Applicable	2	5	0
Education Status by Age			
Any Schooling			
Ages 6–8	45 (93.8%)	42 (89.4%)	33 (97.1%)
Ages 9–10	30 (93.8%)	26 (100%)	43 (100%)
Ages 11–12	19 (95.0%)	27 (100%)	22 (95.7%)
Head of Household Sex			
Male	94	93	94
Head of Household Occupation			
Farmer	39	16	8
Day Labor	29	34	61
Service Holder	15	16	14
Small Businessman	13	32	14
Housewife	2	2	2
Unemployed	2	0	1
Head of Household Occupation by Sex			
Male			
Farmer			
Day Labor	38 (40.4%)	16 (17.2%)	8 (8.5%)
Service Holder	27 (28.7%)	32 (34.4%)	59 (62.8%)
Small Businessman	14 (14.9%)	14 (15.1%)	13 (13.8%)
Unemployed	13 (13.8%)	31 (33.3%)	13 (13.8%)
Female	2 (2.1%)	0 (0%)	1 (1.1%)
Farmer			
Day Labor	1 (16.7%)	0 (0%)	0 (0%)
Service Holder	2 (33.3%)	2 (28.6%)	2 (33.3%)
Small Businesswoman	1 (16.7%)	2 (28.6%)	1 (16.7%)
Housewife	0 (0%)	1 (14.3%)	1 (16.7%)
Unemployed	2 (33.3%)	2 (28.6%)	2 (33.3%)
	0 (0%)	0 (0%)	0 (0%)
Household Monthly Income (Thousands BDT), median (IQR)	20.0 (15.0, 20.0)	14.5 (10.0, 18.0)	10.0 (9.0, 15.0)
Reported Health Conditions in Child			
Asthma	6	1	21
Dengue	1	2	2
Epilepsy	2	1	17
Rash	6	2	48

Values are reported as *n* or *n* (%) unless otherwise specified. Continuous variables such as age, BMI, and arm circumference are shown as mean ± standard deviation (SD). Household income is presented as median (interquartile range, IQR) in thousands of Bangladeshi Taka (BDT). For reference, 1000 BDT ≈ 8.3 USD at the time of data collection.

**Table 2:** Health Measures.

	Children in Three Study Sites (*n* = 300)		
Variable	Barhatta (*n* = 100)	Galachipa (*n* = 100)	Sarankhola (*n* = 100)
Height (cm)	127.2 ± 13.3	125.5 ± 14.2	126.5 ± 10.8
Weight (kg)	23.1 ± 7.7	24.3 ± 7.4	23.5 ± 5.9
PEFR Min-Max, L/min	130–330	80–380	50–390
PEFR Mean (SD), L/min	219.5 ± 45.3	196.4 ± 49.9	234.2 ± 51.9
Blood Lead, Median (Min-Max) (µg/dL)	4.60 (<LOD − 36.4)	1.65 (<LOD − 9.3)	1.65 (<LOD − 12.7)
Hemoglobin Concentration (g %)	12.8 ± 1.0	13.1 ± 1.6	11.0 ± 1.5

Values are shown as mean ± SD or range (min–max), unless otherwise specified. PEFR = peak expiratory flow rate. Blood lead levels are presented as median (min–max). Limit of Detection (LOD) is 3.3 µg/dL. Values re-ported as “Low” by the device were imputed as 1.65 µg/dL for analysis. Blood lead data were available for 99 children in Barhatta.

**Table 3:** Reported Climate and Health-Related Experiences and Perceptions Among Children in Three Study Sites in Bangladesh (*n* = 300).

	Children in Three Study Sites (*n* = 300)		
Exposure Question	Barhatta (*n* = 100)	Galachipa (*n* = 100)	Sarankhola (*n* = 100)
Main Source of Drinking Water			
Shallow	98	0	0
Deep Tubewell	2	99	22
Supplied Water Through Pipe	0	1	5
Crude Pond Water	0	0	13
Refined Pond Water	0	0	15
Rainwater	0	0	38
Other	0	0	7
Closest Place of Healthcare Services			
Government Hospital	0	90	93
Community Clinic	3	9	6
NGO Healthcare Clinic	97	0	0
Village Doctor	0	1	0
Pharmacy	0	0	1
Have Access to Government Healthcare Facility			
District Hospital	0	0	1
Upazila Health Complex	94	100	77
Union Health Center	1	0	17
Community Clinic	5	74	4
Have Health Education Program in School	10	98	93
Have Access to Community Clinic	6	77	8
Faced Any Natural Calamity			
Flood	86	100	40
Drought	11	77	16
Cyclone	1	100	100
Tidal Wave	0	2	29
River Erosion	0	4	7
Ever Became Homeless due to Natural Calamity in Last 10 Years	4	41	78
Heard of Climate Change	32	100	100
Source of Hearing Climate Change			
Newspaper	3	14	2
Radio	0	1	5
Television	15	82	83
Neighbor	6	56	44
Health worker	9	86	30
School teacher	0	59	18
Perceived Type of Climate Change			
Excessive temperature	31	100	93
Excessive cold	0	73	3
Change in pattern of precipitation	1	93	18
Cyclone/Tidal wave	0	27	94
Frequent flood	1	82	3
Water logging	0	2	4
Perceived Reasons for Climate Change			
Deforestation	28	100	97
Industrial effluent	1	37	21
Population growth	3	78	34
Black smoke of vehicles	1	13	46
Excessive carbon emissions to the atmosphere	0	8	4
Quick urbanization and change in livelihood	0	0	3

Distribution of survey responses from the Climate Questionnaire by site (Barhatta, Galachipa, and Sarankhola). All values represent frequency counts (*n*). Categories include water sources, access to healthcare, health education, natural disasters, displacement, and perceptions of climate change and its causes.

**Table 4:** Household Water Salinity and Perceptions by Study Site.

	Children in Three Study Sites (*n* = 300)		
Water Use	Barhatta (*n* = 100)	Galachipa (*n* = 100)	Sarankhola (*n* = 100)
Drinking Water Salinity (ppt)			
25th Percentile	0.10	0.30	0.05
Median	0.20	0.30	0.05
75th Percentile	0.50	0.30	0.06
Cooking Water Salinity (ppt)			
25th Percentile	0.10	0.30	0.05
Median	0.20	0.30	0.05
75th Percentile	0.43	0.30	0.10
Perceived Increase in Local Water Salinity (Past 10 Years)	0	89	89
Use Same Water Source for Drinking and Bathing/Cooking	96	98	46

Values are presented as percentiles (25th, median, 75th) in parts per thousand (ppt) for drinking and cooking water salinity. Observations labeled as “<MDL” were assigned a value of 0.05 ppt, representing half the detection limit. Counts (*n*) are shown for perception-related responses, which were drawn from the Climate Questionnaire.

**Table 5:** School Absenteeism Patterns and Educational Disruptions (Past 30 Days).

	Children in Three Study Sites (*n* = 300)		
Survey Questions	Barhatta (*n* = 100)	Galachipa (*n* = 100)	Sarankhola (*n* = 100)
Days Missed School/Classes			
0 day	34	29	31
1–2 day	22	47	33
3–5 day	5	18	26
6–9 day	1	0	5
10 or more days	37	2	3
Reasons for Missed School			
Personal illness	13	36	48
Family illness	1	16	3
Take care of another person	1	0	2
Work responsibilities	6	1	0
Menstrual cycle	0	0	0
Transportation problems	0	1	14
Weather/Climate emergency	1	12	25
Unexpected school closure	39	0	5
Had to move	6	5	4
Climate Change Affect Education			
Unaffected	58	20	23
Made school unsafe	32	4	17
Impacted journey to school	6	71	61
Impacted teacher	1	4	1
Impacted school facilities	0	8	2
Impacted attendance	1	4	4
Family’s ability to afford schooling	1	0	0

Data from the School and Absenteeism Questionnaire. Values shown as counts (*n*) for days missed, reasons for absence, and perceived climate-related effects on schooling. Children selected one response for days missed, while multiple responses were allowed for reasons for absence and perceived climate-related impacts on schooling.

## Data Availability

The data presented in this study are available on request from the corresponding author due to privacy reasons.

## Supplementary Materials

The following supporting information can be downloaded at: https://www.mdpi.com/article/10.3390/ijerph22111639/s1, Table S1: Distribution of Epilepsy Screen Status by Age Group.

## Abbreviations

The following abbreviations are used in this manuscript:

DCH	Dhaka Community Hospital
EEG	Electroencephalography
GIS	Geographic Information System
SD	Standard Deviation
PEFR	Peak Expiratory Flow Rate
WHO	World Health Organization
