# Supplementary material for: Pediatric Health, Climate Perceptions, and School Absenteeism Across Three Regions of Bangladesh: A Cross-Sectional Study
Source: Int J Environ Res Public Health. 2025 Oct 28;22(11):1639. doi: 10.3390/ijerph22111639 (PMC12652679; doi:10.3390/ijerph22111639)
Supplement: Supplementary file 1 [file ijerph-22-01639-s001.zip › ijerph-3835585-supplementary.pdf]

Supplementary Table

Table S1. Distribution of Epilepsy Screen Status by Age Group.

	**Children in Three Study Sites (*n* = 300)**		
**Epilepsy Screen Status**	**Barhatta (*n* = 100)**	**Galachipa (*n* = 100)**	**Sarankhola (*n* = 100)**
No Epilepsy			
Ages 6-8	48	46	28
Ages 9-10	31	26	35
Ages 11-12	19	27	30
Has Epilepsy			
Ages 6-8	0	1	6
Ages 9-10	1	0	8
Ages 11-12	1	0	3

Number of children (*n*) by age group (6–12 years) and epilepsy screening result. Children were classified as having screened positive for epilepsy if their total score exceeded 1 on the Epilepsy Screening Questionnaire.
